# Antioxidant potential of *Juglans nigra,* black walnut, husks extracted using supercritical carbon dioxide with an ethanol modifier

**DOI:** 10.1002/fsn3.385

**Published:** 2016-05-20

**Authors:** Jonathan Wenzel, Cheryl Storer Samaniego, Lihua Wang, Laron Burrows, Evan Tucker, Nathan Dwarshuis, Michelle Ammerman, Ali Zand

**Affiliations:** ^1^ Kettering University1700 University AveFlintMichigan48504

**Keywords:** Antioxidant activity, black walnut husk, DPPH, ethanol, FRAP, supercritical carbon dioxide extraction, TPC

## Abstract

The black walnut, *Junglas nigra,* is indigenous to eastern North America, and abscission of its fruit occurs around October. The fruit consists of a husk, a hard shell, and kernel. The husk is commonly discarded in processing, though it contains phenolic compounds that exhibit antioxidant and antimicrobial properties. For this study, black walnut husks were extracted using supercritical carbon dioxide with an ethanol modifier. The effects of temperature, ethanol concentration, and drying of walnut husks prior to extraction upon antioxidant potential were evaluated using a factorial design of experiments. The solvent density was held constant at 0.75 g/mL. The optimal extraction conditions were found to be 68°C and 20 wt‐% ethanol in supercritical carbon dioxide. At these conditions, the antioxidant potential as measured by the ferric reducing ability of plasma (FRAP) assay was 0.027 mmol trolox equivalent/g (mmol TE/g) for dried walnut husk and 0.054 mmol TE/g for walnut husks that were not dried. Antioxidant potential was also evaluated using the total phenolic content (TPC) and 1,1‐diphenyl‐2‐picryl‐hydrazyl (DPPH) assays and the FRAP assay was found to linearly correlate to the TPC assay.

## Introduction

Walnut trees grow throughout the temperate regions of the Northern Hemisphere as well as in South America. In the United States, walnut trees are grown for lumber, with the state of Missouri being the leading producer. The fruit of the walnut is commonly harvested and is comprised of the nutmeat, or kernel, the shell, and the husk. The kernel is high in antioxidant compounds and is eaten raw or roasted, or pressed for oil. The shells are used in a variety of applications ranging from abrasives, fillers, thickeners, and dyes. (Mirjalili and Karimi 2013) Walnut husks contain a variety of phenolic antioxidant compounds. (Stampar et al. 2006) Indeed, many components of the walnut tree exhibit antioxidant potential, including the stem, leaf, shell, husk, kernel, and bark. (Yaylaci et al. 2007; Wang et al. 2015) However, unlike the kernel and shell, walnut husks are rarely used commercially and are often discarded. Due to the walnut husks' high phenolic content, the husks have promising antioxidant potential and possibility for valorization.

Due to growing consumer awareness and demand, there is an increasing need for naturally derived chemicals for consumption, cosmetics, pharmaceuticals, and industrial applications. Walnuts contain a variety of beneficial and useful chemical species. Walnuts are a rich source of antioxidants, natural compounds demonstrated to have numerous health benefits. (Vinson and Cai 2012) For example, proanthocyanidins, which are present in high amounts in nuts, help protect the body from sun damage, enhance vision, promote flexibility in joints, arteries and cardiac tissue, and improve blood circulation. (Peralbo‐Molina et al. 2013) Walnut husk extracts have demonstrated antimicrobial effects against gram‐positive bacteria. (Oliveira et al. 2008; Fernandez‐Agullo et al. 2013) Walnut husk extracts can also inhibit xanthine oxidase, an enzyme which forms xanthine which causes hyperuricemia, a metabolic disorder causing inflammation and gout. (Wang et al. 2015) Walnut husks are also high in naphthoquinones, which have a variety of potential uses. Juglone, a naphthoquinone found in walnuts, was demonstrated to have cytotoxic effects against cultured melanoma cells. (Aithal et al. 2009) Naphthoquinones can also be used as broad‐spectrum biocides. (Wright et al. 2007) Black walnuts can also be used as a bio‐herbicide (Shrestha 2009)

The extraction and characterization of walnut husks has not been thoroughly investigated, in particular the black walnut, *Junglas nigra,* which is indigenous to North America. There have been a few studies involving the extraction of the husks of *Juglans regia L*., or the Persian or English walnut, which commonly grows in Eurasia. These studies have focused on extractions using various solvents at ambient pressure. Wang, et al. evaluated the extraction of Persian walnut using a 95% (v/v) ethanol‐water mixture using a mechanical shaker at 45°C. Fernandez‐Agullo et al. evaluated ethanol, methanol, water, and mixtures at room temperature. Akin et al. used methanol and Oliveira et al. utilized boiling water as the extraction solvents. All the investigations used dried walnut husks and employed various means of determining antioxidant potential including the total phenolic content assay (TPC), 1,1‐diphenyl‐2‐picryl‐hydrazyl (DPPH) radical scavenging assay, and ferric reducing ability of plasma (FRAP) assay. However, the use of supercritical fluids for producing walnut husk extracts, either Black or Persian, has not been reported in the literature.

When preparing extracts for the use of pharmaceuticals, cosmetics, and human consumption, it is important to use solvents that are relatively nontoxic, inexpensive, selective, and efficient. Carbon dioxide and ethanol are notable solvents for supercritical fluid extraction that have excellent solvating capabilities and are recognized as safe by the United States Food and Drug Administration. Due to its high diffusivity, supercritical carbon dioxide is extensively used as an environmentally friendly, nontoxic solvent to extract caffeine from coffee and tea and for the extraction of flavors and essences. (DeSimone 2002) However, at moderate pressures, supercritical carbon dioxide is relatively nonpolar, which presents a challenge when extracting antioxidant compounds that are polyphenolic and slightly polar. (Leeke et al. 2005) To take advantage of supercritical carbon dioxide's high diffusivity and low critical temperature, 31.1°C, ethanol may be added as a modifier to increase the mixture's polarity. The use of ethanol in supercritical carbon dioxide has been demonstrated to improve the extraction of antioxidants from grape pomace. (Casas et al. 2010; Dalmolin et al. 2010; Oliveira et al. 2013)

The purpose of this study was to use supercritical carbon dioxide with ethanol as a modifier, or cosolvent, to extract antioxidants from the husks of *Junglas nigra*, or black walnut. Ethanol was chosen as a cosolvent to improve the extraction of antioxidants. Both temperature and ethanol content were varied to determine an optimal extraction condition for increasing the antioxidant potential of the extract. Furthermore, the effect of drying the walnut husk prior to extraction upon antioxidant potential was also of interest, since decreasing drying could result in decreased energy‐use. This study was unique in that while there are articles on supercritical fluid extraction of Persian walnut kernels (Crowe et al. 2002; Salgin and Salgin 2006; Martinez et al. 2008), there is a lack of references on supercritical fluid extraction of walnut husks, in particular black walnut.

## Materials and Methods

### Chemicals

The following reagent grade or greater chemicals were used: concentrated hydrochloric acid, ferric chloride hexahydrate, trolox, and 2,4,6‐tris(2‐pyridyl)‐s‐triazine (TPTZ) from Sigma Aldrich; ACS certified grade ferrous sulfate heptahydrate, glacial acetic acid, molecular biology grade ethanol (200 proof), and DPPH from Fisher Scientific; sodium acetate trihydrate (≥99%) from Avantor; gallic acid monohydrate from Acros Organics; Folin‐Ciocalteu reagent from Merck; sodium carbonate monohydrate from J. T. Baker Chemical; and ultrapure water produced on‐site. Nitrogen, 99.998% and helium, 99.999% were from Praxair. Chemicals were used without further purification. All chemicals originated from the United States.

### Plant material

Black walnuts, *Junglas nigra,* were obtained in southeastern Michigan, United States, at the end of the 2013 growing season. Fallen walnuts were retrieved while the husks were still green. Immediately following retrieval, the husks were manually removed from the shell. Husks were either frozen or dried. Husks that were frozen, were frozen in a nitrogen‐purged container at −20°C for several days, then ground while frozen in a coffee grinder and stored in a nitrogen‐purged container at −20°C until time of use. Husks that were dried, were dried in a vacuum oven at 55°C and −84 kPa gauge for 72 h. Following drying, the husks were ground in a coffee grinder and stored in a nitrogen‐purged container at −20°C until time of use. The particle size of the ground husks was ranged from 20 to 50 mesh.

### Moisture content

The moisture content of the walnut husk was determined by the following method. Approximately 2.000 g of frozen walnut husk was weighed in a circular aluminum drying pan. The sample was dried under vacuum, −84 kPa gauge, at 55°C in a Fisher Scientific^™^ Isotemp^™^ Model 281A vacuum oven (United States). Suction was created by a Fisher Scientific^™^ Maxima C Plus vacuum pump. After approximately 19 h, the sample was removed from the oven and reweighed. The moisture content was measured using five different samples prepared on different days. The moisture content was determined by the following formula, (*M*_wet_−*M*_dry_)/*M*_wet_ × 100%.

### Extraction system

**Figure 1:**
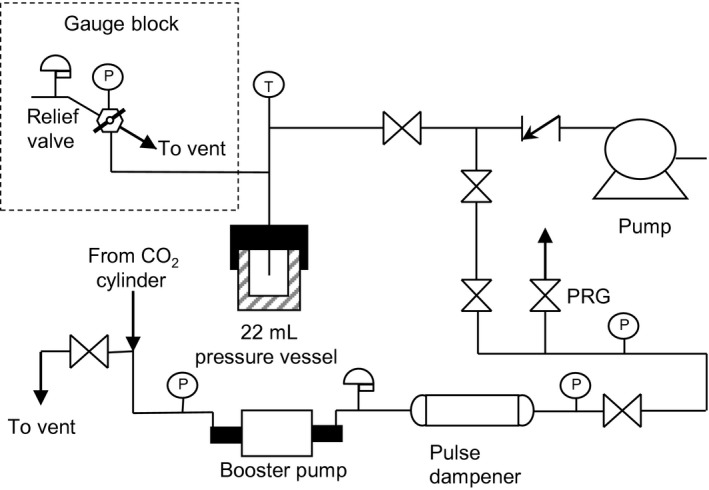
Process flow diagram for the batch extraction system modified with gas delivery system.

Extractions were performed in batches in a modification of a previously described custom‐built batch extraction system designed to heat liquids to supercritical fluid conditions with a total volume of 24 mL, Figure 1. (Wenzel et al. 2015) The modification was the addition of a gas delivery system consisting of a two‐stage, pneumatically driven gas booster pump, Haskel International, model 80875‐DP‐28881 that supplied carbon dioxide at cylinder pressure. Following the booster pump, pressurized carbon dioxide entered a 1 L double‐end sample cylinder with a pressure rating of 5000 psig at room temperature, Swagelok, 316L‐50DF8‐1000. The sample cylinder acts as a pulse dampener. All shut‐off valves for the gas delivery system are two‐way straight valves with a regulated stem, Autoclave Engineers part number SW4081. These valves were used as purge valves (PRG), and shutoff valves. Pressure at the discharge of the gas delivery system was indicated by an Ashcroft 4 ½” diameter Bourdon tube pressure gauge with a range 0–5000 psig (0–346 bar) and reading increments of 20 psi (1.4 bar). Briefly, the extraction system consisted of a heated pressure vessel, pressure gauge block with relief valve, thermocouple with indictor, and a pump with purge system. The pressure vessel was a 22 mL bolt‐closure pressure vessel was a Parr Instruments Company (Moline, IL) model 4742. The pressure vessel was heated by submersion in a water bath heated by a hot plate.

### Extraction method

Prior to use, the extraction system was scoured, pressurized with acetone, then purged and dried with nitrogen. The experiments varied in temperature, ethanol weight fraction, and pretreatment of the walnut husk, either dried or not. Overall system density was held constant with a target density of 0.75 g/mL and pressure was allowed to vary with temperature. The density was calculated using Aspen Process Simulation software using the Peng–Robinson Equation of State with Wong–Sandler mixing rules. For each experiment, approximately 0.50 g of walnut husk, dried or not dried, were weighed and placed into the pressure vessel. In addition, a predetermined amount of ethanol was placed into the pressure vessel. Prior to use, ethanol was purged of oxygen by bubbling nitrogen gas for 15 min. The pressure vessel was then purged with nitrogen, sealed, and connected to the batch extraction system. It was heated to the target temperature and then carbon dioxide was fed into the pressure vessel until the target pressure was reached. Then the pressure vessel was held at a constant temperature for 60 min. Next the pressure vessel was allowed to cool, opened, and the liquid extract suctioned out using a transfer pipette. Without posttreatment, the liquid extract was stored in a glass vial blanketed with nitrogen, double‐sealed, in the dark at 4°C to protect the sample from decomposition. Prior to analysis, the extract was centrifuged at 13,800 g for 5 min in a cold microcentrifuge; only the supernatant was utilized for the assays. The extracts were analyzed using FRAP, TPC, and DPPH assays.

### FRAP assay

The Ferric Reducing Ability of Plasma (FRAP) assay was performed as previously described, (Benzie and Strain 1996) where the absorbance of the analytes were measured at 590 nm and 37°C with a Cary 300 Bio UV/Vis Spectrophotometer. The FRAP reagent was prepared by mixing aliquots of 25 mL of 0.3 mol/L acetate buffer, 2.5 mL of a solution of 10 mmol/L TPTZ and 40 mmol/L HCl, and 2.5 mL of 20 mmol/L FeCl_3_ together. FRAP reagent was prepared on the day of measurement and maintained at 37°C once prepared. A calibration curve for Fe^2+^ was generated by mixing 900 *μ*L FRAP reagent, 90 *μ*L ultrapure water, and 30 *μ*L of FeSO_4_ solutions ranging from 100 to 2500 *μ*mol/L of FeSO_4_·7H_2_O Ferrous sulfate gives a change in absorbance that is double an equivalent Trolox molar concentration. (Benzie and Strain 1996) Extract samples were assessed by mixing 900 *μ*L FRAP reagent with 116 *μ*L ultrapure water and 4 *μ*L of diluted sample extract; each sample extract was diluted 1:4 in ethanol to keep the final absorbance within range of the calibration curve. All calibration standards and samples were referenced to a blank solution of FRAP reagent. Sample readings were assessed at 10 min and averaged with standard deviation. Each extract was analyzed at least in triplicate on two different days using the FRAP assay. The antioxidant potential for each extract was reported as an average and standard deviation of these measurements. Average extract sample absorbance values were subtracted by the absorbance due to the color of the sample to normalize the data. Corresponding Fe^2+^ concentrations generated by the samples were determined by the calibration curve and converted to mmol Trolox equivalent/gram of walnut husk (mmol TE/g).

### Total phenolic content

The total phenolic content (TPC) or folin‐ciocalteu (F‐C) assay, determined the oxidation of phenolic compounds by a molybdotungstate reagent yielding a colored product with *λ*_max_ at 745–750 nm. (Folin and Ciocalteu 1927; Singleton and Rossi 1965) The TPC assays of the extracts were carried out using a previously described procedure utilizing a 96‐well microplate. (Ainsworth and Gillespie 2007) Ethanol solutions of gallic acid ranging from 50 *μ*mol/L to 1000 *μ*mol/L were used as the standards to create the calibration curve. The samples were diluted between 1:10 and 1:40 in ethanol to keep the final absorbance within range of the calibration curve. The absorbance at 765 nm was measured after 120 min of incubation at 25°C using a BioTek Synergy HT microplate reader. Total phenolic content was determined as mg of gallic acid equivalent/g of walnut husk (mg GAE/g). Each extract was analyzed by the TPC assay in duplicate on two different days.

### DPPH assay

The antioxidant activity was also measured in terms of DPPH radical scavenging capacity using a previously described kinetic DPPH assay. (Cheng et al. 2006) A 1.04 mmol/L DPPH in ethanol stock solution was prepared then stored in a foil‐wrapped, nitrogen‐purged, conical tube in a 4°C refrigerator. The DPPH stock solution was diluted with ethanol to 0.208 mmol/L. A six‐point calibration curve was produced using standard solutions of Trolox in ethanol; Trolox concentrations ranged from 10 to 85 mmol/L. All tested extract samples were diluted in ethanol such that the final absorbance was within range of the calibration curve. The assay was performed in a 96‐well polystyrene plate, and the final volume in each well was 200 *μ*L. DPPH controls, four per plate, were created by mixing 100 *μ*L of ethanol with 100 *μ*L of 0.208 mmol/L DPPH solution, and ethanol blanks, four per plate, were created with 200 *μ*L ethanol. The method by Cheng et al. was modified to include sample controls for color correction; one control per sample was created with 100 *μ*L of diluted extract combined with 100 *μ*L of ethanol. Next, 100 *μ*L of diluted sample or a Trolox standard solution was added to wells in duplicate, generating the calibration curve or sample readings. Then, 100 *μ*L of 0.208 mmol/L DPPH solution was added to each sample or standard well simultaneously using a multichannel pipette. Upon addition of DPPH, the plate was immediately read for absorbance at 515 nm for 40 min at 1 min intervals at 25°C using a BioTek Synergy HT microplate reader.

The absorbance data were converted to %‐DPPH quenched versus time by applying the following equation:

$$\%\text{DPPH quenched}=\left(1-\frac{A_{\mathrm{sample}}-A_{\mathrm{blank}}}{A_{\mathrm{control}}-A_{\mathrm{blank}}}\right)\times 100$$

where A_sample_ was the absorbance of the diluted sample or standard, A_blank_ was the absorbance of the 200 *μ*L ethanol blank, and A_control_ was the absorbance of the DPPH control which is 100 *μ*L DPPH solution with 100 *μ*L ethanol. Each curve was then integrated using the trapezoid rule from 0 to 40 min to find the area under the curve (AUC). For the calibration curve, AUC was plotted against Trolox concentration; a linear regression equation with *R* ^2^ = 0.99 was obtained from the calibration curve and used to convert AUC of each sample extract dilution to Trolox equivalents. Reported values for each extract were the average of at least two DPPH assays run on separate days for a minimum of four replicate analyses of each extract.

### Experimental design and statistical analysis

Two factors that are important in supercritical carbon dioxide extraction using an ethanol modifier, temperature, and ethanol content, were evaluated using a 2^2^ factorial design with three replicates for the center point. The responses for the factorial design, FRAP and TPC, were evaluated by analysis of variance (ANOVA) tests at a 0.05 level of significance. The range for temperature was 50–68°C and for ethanol content was 10–20 wt‐%. The total density of the system was held constant at 0.75 g/mL as estimated using the Peng–Robinson Equation of State with Wong–Sandler mixing rules. Dried walnut husk was evaluated using the factorial design with center points. Wet, or as‐is, walnut husk was also evaluated using the factorial design; however, due to lack of material, there were an insufficient number of replicates to perform an ANOVA test. Statistical analysis was performed using Minitab^®^ version 16.2.0 statistical analysis software (State College, Pennsylvania, USA). At least three independent analyses for FRAP, TPC, and DPPH were performed for each extraction sample. All experimental results were reported as mean values with corresponding standard deviations of assay measurements. A *P*‐value less than 0.05 was considered statistically significant.

## Results and Discussion

Supercritical carbon dioxide with ethanol as a cosolvent was used to produce extracts of the husks of *Junglas nigra,* or black walnut husks. For supercritical fluids, the solvating power can be influenced by changes in temperature, density, and cosolvent content. For this study, the effects of extraction temperature and ethanol content were varied and for simplicity, the total solvent density of the extraction system was held constant at 0.75 g/mL, which permitted the pressure to remain within the maximum allowable working pressure of the extraction system. Temperature ranged from 50 to 68°C and ethanol content from 10 to 20 wt‐%. In addition, the effect of drying the walnut husks prior to extraction was also characterized. Walnut husks were either extracted as‐is with naturally occurring moisture content or were thoroughly dried prior to the extraction. The moisture content of the walnut husk prior to drying was found to be 69.6 ± 1.2% water by weight.

The effects of temperature and ethanol content of the extraction solvent upon the antioxidant potential of dried walnut husk extracts was evaluated using a 2^2^ factorial experimental design with three replicates for the center point. The antioxidant potential for the factorial experimental design was measured using the FRAP assay and the TPC assay. The effects of temperature and ethanol content upon antioxidant potential of dried and wet, or as‐is, walnut husk upon FRAP and TPC are shown in Table 1. The maximum antioxidant potential for dried walnut husks was 0.0270 ± 0.0017 mmol TE/g as measured by the FRAP assay and 4.06 ± 0.16 mg GAE/g as measured by TPC, which was extracted at the maximum temperature of 68°C and ethanol content, 20 wt‐%. The minimum antioxidant potential for dried walnut husks extracts was at the minimum conditions of 50°C and 10 wt‐% ethanol, with 0.0071 ± 0.0001 mmol TE/G for FRAP and 0.97 ± 0.05 mg GAE/g for TPC. As temperature and ethanol content increase, the antioxidant potential of the dried walnut husk extracts increase. Wet walnut husk extracts antioxidant potential was between 2 and 10 times the antioxidant potential of dried walnut husk extracts produced at similar extraction conditions.

**Table 1:** Antioxidant potential as measured by the FRAP and TPC assays of wet and dried walnut husks extracted with supercritical carbon dioxide with an ethanol modifier

T (°C)	EtOH wt‐%	Dried walnut husk		Wet walnut husk	
		FRAP (mmol TE/g)	TPC (mg GAE/g)	FRAP (mmol TE/g)	TPC (mg GAE/g)
50	10	0.0071 ± 0.0001	0.97 ± 0.05	0.0710 ± 0.0022	7.63 ± 0.20
68	10	0.0153 ± 0.0003	2.52 ± 0.06	0.0537 ± 0.0014	7.50 ± 0.24
50	20	0.0213 ± 0.0009	2.73 ± 0.04	0.0390 ± 0.0011	5.48 ± 0.06
68	20	0.0270 ± 0.0017	4.06 ± 0.16	0.0612 ± 0.0022	9.17 ± 0.20
60	15	0.0133 ± 0.0004	1.99 ± 0.02	0.0554 ± 0.0019	7.13 ± 0.24
60	15	0.0128 ± 0.0005	1.99 ± 0.08		
60	15	0.0135 ± 0.0010	2.35 ± 0.08		

FRAP, ferric reducing ability of plasma; TE, trolox equivalent; TPC, total phenolic content.

**Figure 2:**
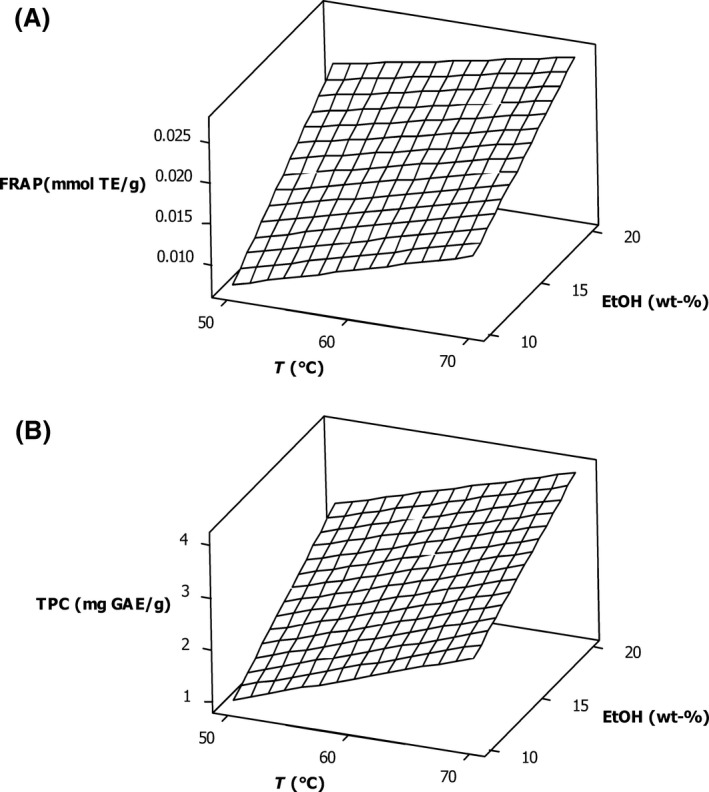
Response surface analysis for the effect of temperature and ethanol content upon (A). FRAP antioxidant potential for dried walnut husk, (B). TPC for dried walnut husk.

**Figure 3:**
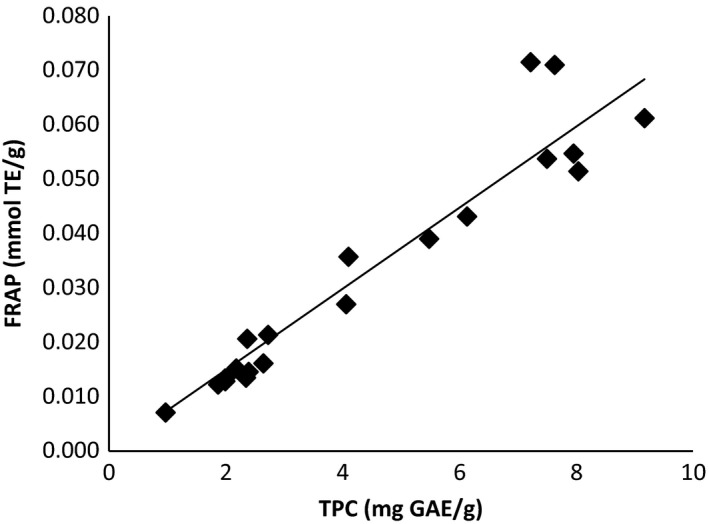
Correlation between TPC and FRAP of dried and wet walnut husk extracts (*y* = 0.0075*x*,* R* ^2^ = 0.9112), 0.5 g walnut husk, total density 0.75 g/mL, and hold time of 60 min.

The analysis of variance of the effect of temperature and ethanol content upon the antioxidant potential as measured by the FRAP and TPC assay for dried walnut husk extracts is shown in Table 2. The main effects, temperature and ethanol weight fraction in carbon dioxide, upon total phenolic content as measured by the TPC assay and upon antioxidant potential as measured by the FRAP assay, were statistically significant with *P* ≤ 0.05. While the two‐way interaction between temperature and ethanol was not statistically significant for FRAP and TPC, a response surface analysis of the data is shown in Table 1 may still provide some insight into optimal processing conditions. The lack of significance may be due to variability in performing the extraction, as well as the FRAP and TPC assays. The increase in the *P*‐value for the TPC assay, compared to the FRAP assay, may be due to an outlier in the final reported replicate experiment for TPC shown in Table 1. The response surfaces for both FRAP and TPC are shown in Figure 2. FRAP and TPC data had a linear correlation with *R* ^2^ = 0.9112 shown in Figure 3. As temperature and ethanol content of the extraction system is increased, the antioxidant content of the dried walnut husk extracts increased.

**Table 2:** ANOVA of the effects of temperature and ethanol weight fraction upon antioxidant potential for dried walnut husk

Source	DF	P_FRAP_	P_TPC_
Main effects	2	0.001	0.017
T (°C)	1	0.002	0.020
EtOH (wt‐%)	1	0.001	0.015
2‐Way Interactions	1	0.061	0.658
T (°C)*EtOH (wt‐%)	1	0.061	0.658

The extraction of antioxidant compounds from plant matter is expected to increase with increasing temperature, as long as the temperature is not excessive which could lead to decomposition of the compounds. To discuss and compare the effect of temperature in extraction using pressurized fluids upon antioxidant potential, the extraction of antioxidants from grape seeds and pomace may be considered. In one study involving grape seed extraction using supercritical carbon dioxide with an ethanol modifier, antioxidant activity, as well as total phenols, increased when extraction temperature increased from 37 to 46°C, which falls within the temperature range of the walnut husk study. (Ghafoor et al. 2012) For extraction of grape pomace using superheated liquid water extraction, which is extraction with heated, pressurized liquid water, antioxidant activity likewise increased as the temperature increased from 100 to 140°C. (Aliakbarian et al. 2012) Similar trends were observed in other studies evaluating the extraction of wine‐making wastes using supercritical carbon dioxide with an ethanol modifier. (Pinelo et al. 2007; Casas et al. 2010) As the temperature increases, the fibrous materials in the walnut husk likely begin to relax and the diffusivity of the extraction fluid increases, resulting in an increase in antioxidant compounds extracted. This would result in correspondingly higher TPC and FRAP values.

To extract slightly polar compounds using supercritical carbon dioxide, typically a polar modifier is added, and for this study, ethanol was used. As the amount of ethanol increased, the antioxidant potential increased for dried walnut husk extracts shown in Figure 2. In supercritical carbon dioxide, some of the characteristics of the fluid change as the amount of ethanol increases, such as an increase in the polarity of the extraction fluid. Since phenolic compounds are slightly polar, the increase in polar characteristics of the extraction solvent will result in a higher antioxidant content in the extract; “like dissolves like.” The trade‐off is that by increasing the ethanol content, the critical pressure and temperature of the mixture also increases.(Pohler and Kiran 1997) A similar increase in antioxidant potential and total phenolic content was also observed in the supercritical carbon dioxide extraction of both strawberries and grape seeds as ethanol content increased from 0 to 20%. (Murga et al. 2000; Akay et al. 2011)

For both TPC and FRAP analysis, the highest antioxidant potential was observed at an extraction condition of 68°C with 20 wt‐% ethanol. It may be possible that even higher temperatures and ethanol content can result in higher antioxidant potential extractions, however, there are practical concerns as more extreme conditions are reached. First, if a higher density extraction solvent is desired for the extraction, if the temperature is increased, the extraction pressure must also increase. In addition, the higher the extraction temperature, the greater the likelihood that the antioxidant compounds will decompose. Furthermore, adding more ethanol increases the carbon dioxide‐ethanol critical point, which increases the operating temperature and pressure if the carbon dioxide‐ethanol mixture is to remain supercritical. While carbon dioxide's critical point is 31.1°C at 73.8 bar, the critical point of pure ethanol is 241°C at 63 bar. In addition, due to nonideal fluid behavior, the mixture's critical point is not linear in relationship with the weight fraction of ethanol. The critical pressure of carbon dioxide‐ethanol reaches a maximum of over 150 bar at 35 wt‐% ethanol. (Pohler and Kiran 1997) Increased temperature and pressure would require additional energy for heating and pumping, as well as heavier walls for the pressure vessel in which the extraction takes place.

**Figure 4:**
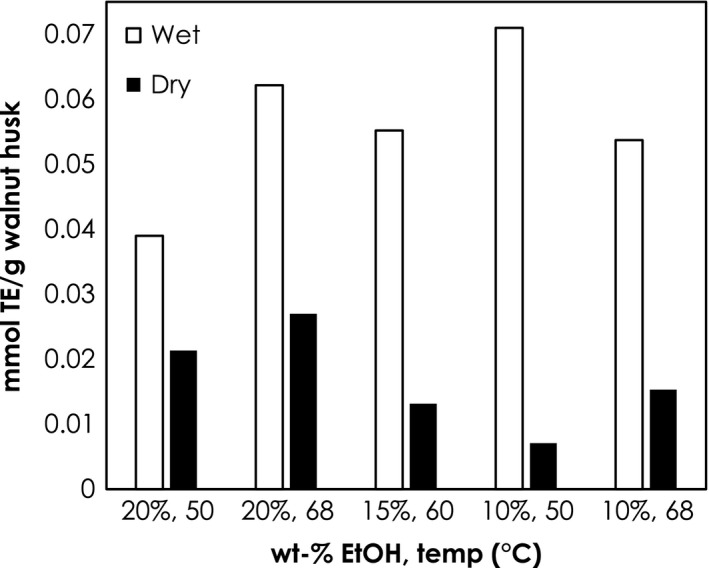
Comparison of FRAP antioxidant potential between dried and damp walnut husk from 50 to 68°C and 10 to 20 wt‐% ethanol.

Quite frequently in extraction studies, plant matter is dried postharvest and stored in a controlled‐environment prior to extraction. This has been done in for a variety of extraction studies involving berries, such as strawberry, chokeberry, and grapes, leaves of berry crops, and nuts, such as walnuts. (Oliveira et al. 2008, 2013; Akay et al. 2011; Pieszka et al. 2015) For walnut husk extraction, an extensive literature review reveals that investigators commonly dry the walnut husks at various temperatures prior to extraction, Table 3. There are many reasons to dry plant matter: to prevent spoilage for better storage until time of use, to concentrate the chemicals in the plant matter, or to control the variability that moisture may present. However, for walnut husks extracted in this study, using supercritical carbon dioxide with an ethanol modifier, postharvest drying adversely effects the antioxidant potential of the extract, seen in Figure 4. The walnut husks that are immediately frozen at −20°C postharvest then ground, but not dried prior to extraction, resulted in extracts that have double to 10‐fold the antioxidant content of walnut husk extracts produced from dried walnut husks. This is surprising since it would be expected that the antioxidant content would have been concentrated in the dried walnut husk. However, there are a couple of possibilities for the increase in antioxidant potential that was observed between dried and wet walnut husk. It is possible that while drying, as the water wicked out of the walnut husk, it extracted the antioxidant compounds out *in situ* and then the antioxidants were deposited on the drying tray prior to the water evaporating. Another possibility is that the water in the walnut husk facilitates the extraction using carbon dioxide and ethanol, since water is polar. It is also conceivable that the elevated drying temperature of 55°C for 72 h may have resulted in the decomposition of some of the antioxidant compounds in the walnut husks. Loss of antioxidant potential has been observed when drying temperatures are elevated from 40 to 70°C for peppermint leaves and motherwort (Yi and Wetzstein 2011) Increasing drying temperature also causes loss of phytochemicals from *Echinacea angustifolia*. (Maggini et al. 2010)

**Table 3:** Comparison of total phenolic content of walnut husk extracts by various reported methods

Investigator	Material	Pretreatment	Extraction method	TPC
This work	Junglas nigra Husk, 2013, US	Frozen, dried ground 72 h, extracted	Supercritical carbon dioxide with ethanol modifier	0.97–4.06^a^
This work	Junglas nigra Husk, 2013, US	Frozen, ground, extracted	Supercritical carbon dioxide with ethanol modifier	5.48–9.17^a^
(Tabaraki and Rastgoo 2014)	Juglans regia L. Husk, 2010, Iran	Dried at 40°C for unknown time, ground, frozen	Ultrasonic‐assisted extraction for 30–70 min, 30–60°C, 45–65% ethanol in water	6.28–7.23^a^
(Oliveira et al. 2008)	Juglans regia L. Husk, 2006, Portugal	Frozen, freeze dried, ground	Boiling Water for 45 min	32–74.08^b^
(Wang et al. 2015)	Juglans regia L. Husk, 2013, China	Dried at unknown time and temperature, ground, frozen	95% (v/v) ethanol using a mechanical shaker for 4 h at 45°C	0.0874^a^
(Fernandez‐Agullo et al. 2013)	Juglans regia L. Husk, year not given, Portugal	Frozen, freeze dried, ground	Ethanol, methanol, ethanol‐water, methanol‐water mixtures at room temperature for 45 min	51.87–84.46^b^
(Akin et al. 2013)	Juglans regia L. Husk, 2011, Turkey	Dried at room temperature for a week, frozen	Methanol, 2–8 h, temperature not given	26.21–29.50^b^

TPC, total phenolic content

a mg GAE/g husk

b mg GAE/g extract

Literature providing a systematic study of the extraction of black walnut, *Junglas nigra,* husks is scarce as is the use of supercritical fluids to produce extracts of the husk of any species of walnut. Most evaluations of walnut husk extractions involve the Persian, or English, walnut, *Juglans regia* L.*,* and make use of fluids at ambient pressures. Comparing other extraction methods is challenging, not only due to the difference in species of walnut, but also due differences in drying and lack of uniformity in reporting FRAP and TPC values. Extraction of the husk of *Juglans regia L*. was evaluated by other investigators using boiling water, heated ethanol with a mechanical shaker, alcohols and alcohol‐water mixtures at room temperature, and ultrasonic‐assisted extraction over various extraction times, and drying conditions of the husks, listed in Table 3. Investigators either reported TPC in terms of either gallic acid equivalent per gram of husk or gallic acid equivalent per gram of extract. There is no direct means of comparison between these reporting methods. TPC reported in terms of gallic acid equivalent of extract will be significantly higher than gallic acid equivalent per gram of husk since extracts due to concentration of the antioxidants. When comparing TPC results using the same reporting method, ultrasonic‐assisted extraction of Persian walnut husk using ethanol and water, by Tabarkai et al. had comparable values to wet, black walnut husk extracts from this work. Differences in the ultrasonic‐assisted extraction and supercritical fluid extraction include drying temperature and reaction time for the TPC assay. In comparison to supercritical fluid extracted walnut husks, conventional extraction using ethanol with a mechanical shaker for Persian walnuts by Wang et al. resulted in a 50‐fold decrease in total phenolic content. Both supercritical fluid extraction and ultrasonic‐assisted extraction are a means of overcoming resistance to mass transfer in the desorption and diffusion of chemical species. Ultrasonic‐assisted extraction makes use of low‐frequency energy to facilitate agitation and mass transfer; supercritical fluid extraction makes use of supercritical fluid's higher diffusivity as compared with liquids. Another method employed to overcome mass transfer resistance is vigorous agitation. Further study, including identical species of walnut and drying method is needed to determine the effectiveness of ultrasonic‐assisted extraction compared to supercritical fluid extraction depending upon the desired end use of the extract. The differences in the two methods may result in preferential selectivity of particular chemical species for one method over the other.

**Figure 5:**
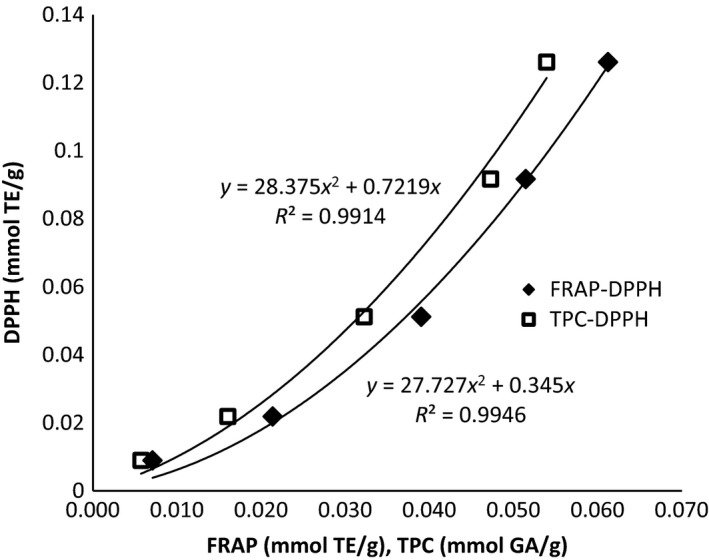
Correlation between TPC and FRAP with respect to DPPH of dried and wet walnut husk extracts, 0.5 g walnut husk, total density 0.75 g/mL, and hold time of 60 min. DPPH, 1,1‐diphenyl‐2‐picryl‐hydrazyl.

Finally, a random sample of three extracts from dried walnut husk and three extracts from damp walnut husks were evaluated using the DPPH radical scavenging assay. All walnut husk extracts evaluated were found to inhibit the DPPH radical to various extents, with activity ranging from 0.006 to 0.054 mmol TE/g. DPPH radical scavenging capacity was correlated with the both FRAP antioxidant potential and TPC shown in Figure 5. Both FRAP and TPC correlated with DPPH activity with a second‐order polynomial fit with an *R* ^2^ = 0.99. Persian walnut husk also shows inhibition of DPPH radical scavenging.(Akin et al. 2013; Fernandez‐Agullo et al. 2013; Tabaraki and Rastgoo 2014).

## Conclusions

This study demonstrates, for the first time, the extraction of antioxidants from the husk of *Junglas nigra*, or black walnut. This study also demonstrates, for the first time, the use of supercritical fluid extraction using carbon dioxide with an ethanol modifier in the extraction of walnut husks. The extraction method, when applied to walnut husks demonstrated high total phenolic content when compared with conventional extraction and similar total phenolic content when compared with ultrasonic‐assisted extraction using ethanol and water. The maximum antioxidant potential as measured by the FRAP assay and highest TPC values were measured at the highest temperature, 68°C and the highest ethanol content, 20 wt‐%.

## Conflict of Interest

None declared.
